# The geometric approach to human stress based on stress-related surrogate measures

**DOI:** 10.1371/journal.pone.0219414

**Published:** 2021-01-25

**Authors:** Petr Kloucek, Armin von Gunten

**Affiliations:** Department of Psychiatry, Lausanne University Hospital, Lausanne, Switzerland; Bruno Kessler Foundation, ITALY

## Abstract

We present a *predictive Geometric Stress Index* (pGSI) and its relation to behavioural Entropy (bE). bE is a measure of the complexity of an organism’s reactivity to stressors yielding patterns based on different behavioural and physiological variables selected as Surrogate Markers of Stress (SMS). We present a relationship between pGSI and bE in terms of a power law model. This nonlinear relationship describes congruences in complexity derived from analyses of observable and measurable SMS based patterns interpreted as stress. The adjective geometric refers to subdivision(s) of the domain derived from two SMS (heart rate variability and steps frequency) with respect to a positive/negative binary perceptron based on a third SMS (blood oxygenation). The presented power law allows for both quantitative and qualitative evaluations of the consequences of stress measured by pGSI. In particular, we show that elevated stress levels in terms of pGSI leads to a decrease of the bE of the blood oxygenation, measured by peripheral blood oxygenation S_*p*_O_2_ as a model of SMS.

## 1 Introduction

This paper is an extension of our previous spectral theory of human stress, [1], providing a posteriori analysis of stress experienced by a human subject (cf. Section 4.1). In the presented communication, we explore the possibility to predict mental and physical stress based on a finite number of measurements using various types of mathematical techniques (cf. Section 3.8).

Continuous psychological stress monitoring in daily life is important. Chronic stress has become a central issue in clinical and economic contexts of modern societies as a result of its deleterious impact on both physical and psychological health [2–4].

Thus, continuous psychological stress monitoring in these contexts may become an important aid to monitor stress levels in various contexts. Stress monitoring may allow for early detection or prediction and, thus, prevention of stress-related health problems [5].

There are two conventional methods to measure psychological stress, i.e., self-reported and body fluid analysis. The self-report method is hard put to monitor human stress consistently due to the lack of consistency. The body fluid analysis is invasive and does not measure stress continuously.

We intend to illustrate the potential of complexity analytics using physiological and behavioural data of a few healthy subjects. Specifically, we decided to use heart frequency variability (HRV) and step frequency (SF) for the analyses as predictor variables of the oxygen saturation in the blood as a binary perceptron approximated by S_*p*_O_2_. These variables will serve as surrogate markers of stress (SMS). We computed the heart rate variability from heart rate.

Other approach based on the Vector Support Machine is reported in [6] The purpose of this communication is fourfold.

*First*, we introduce the *predictive Geometric Stress Index* using HF, SF and S_2_O_2_. Complexity plays an important role in the objective indexing of SMS patterns [1, 7]. We use the adjective “geometric” to indicate that we compute separation curve(s) in the (0, 1)^2^ complexity space given the complexity projections of HRV × SF based on the respective values of the perceptron separating normoxemia domain(s) from hypoxemia domain(s).

*Second*, we re-define bE we have proposed elsewhere [8]. bE measures behavioural and/or physiological reactivity distribution of a sequence of different events represented by the complexity of a single pattern corresponding to, e.g., HRV. The concept resides with the assumption that bE should *not* be evenly, or nearly so, distributed in time. This approach is similar to the entropy concept in the physics measuring uneven distributions of energy among atoms. Increasing non-uniform energy distribution increases the entropy of “a non-organic system” while keeping its complexity high. Similar argumentation can be applied to living organisms [9].

*Third*, we introduce the *predictive Stress Resistance Index* (pSRI) that is meant to quantify human resistance to various forms of stress. pSRI is based on perceptron values and their distances to the separation hyperplanes yielded by analyses of time-series of SMS.

*Fourth*, we propose a power law model linking pGSI with behavioral entropy applying it to time-series of SMS. We aim to predict stress in terms of pGSI in human subjects as measured through the evolution of complexity patterns, using a power law relating pGSI and bE(.).

In short, we present a proof of concept study showing that complexity analysis of HRV and SF and S_*p*_O_2_ can be used as SMS to predict human stress.

## 2 Materials and methods

### 2.1 Quantities measured and computed

- Heart Frequency (HF) / Heart Rate Variability (HRV)
  Heart Frequency was estimated by means of a motion-compensating algorithm from pulse-induced variations of optical reflection from the skin under the sensor.
  Heart rate variability (derived and computed from HF) refers to the variation in the time interval between any two heartbeats. It is controlled by the autonomous nervous system, which is very sensitive to stress. We can, thus, use HRV as a surrogate marker of stress. It may be conceptualized as the standard deviation of the mean heart rate over a certain period of time.
- Steps Frequency (SF)
  Movement corresponds to the instantaneous whole-body activity of a human subject. The measurements were performed with a 3-axis accelerometer. The indicator is given by energy variations of low-passed filtered differentials of accelerometer measurements. Steps Frequency was determined as the inverse of speed of movement.
- Blood Oxygenation Approximation (S_*p*_O_2_)
  S_*p*_O_2_ was measured using reflected red and infrared light supported by motion-compensating algorithms to estimate the ratio of hemoglobin molecules in arterial blood.

### 2.2 Quantities used for the purpose of the study

We used HRV and SF for the analyses as predictor variables and S_*p*_O_2_ as a binary perceptron. The choice of HRV and SF is meant to allow distinguishing increased physical activity (e.g. sport activity leading to congruent increase of HF and SF) from mental stress leading to incongruent HF increase and SF decrease. We felt the limitation to two variables was adequate mainly for two reasons.

First, HR (usually its variability) is often used as an indicator of stress, and SF is a reasonable indicator for the intensity of physical activity. Second, we felt the use of only few variables was appropriate for the sake of simplicity for this proof of concept. With the purpose to introduce an element of prediction, we assume that the choice of S_*p*_O_2_ as a binary perceptron is adequate. Preliminary analysis revealed that S_*p*_O_2_ as measured over time showed a great variability lending itself for the purpose of complexity analyses. Furthermore, the term hypoxemia as used in this paper refers to lower levels of oxygenation relative to the mean oxygen saturation of its complexity. A further reason for the choice of the three SMS is that each of them has much bigger variance over time compared to the rest of the sensory data we had at our disposal (cf. also later).

#### Surrogate markers of stress

Stress is a complex phenomenon with numerous expressions that can be in the realm of subjectivity, cognition, emotion, behaviour, impact etc. We cannot measure stress per se. We can only measure one or more expressions of stress. Furthermore, we can analyze the measures of stress expressions in appropriate ways. We call the results of these analyses or the measures of these expressions “surrogate markers for stress”(SMS). Thus, for instance, data delivered by sensors, such as HRV or SF, are projected onto a three-dimensional space with the third dimension being the S_*p*_O_2_ perceptron. The combination and its distribution is what we interpret as SMS.

### 2.3 Procedures

The Biovotion VPM multi-sensor system was used. Each subject fixed the device to the left arm using an adaptable elastic band according to the instructions established by Biovotion, SA.

They carried the device for at least 6 hours between 13pm and 19pm while they went on with their usual activity once at their work place and once during a weekend day depending on their own choice. However, they were instructed to choose a day with varying activity, preferably including physical activities and intermittent resting. The subjects were to fill in a prepared standard sheet to jot down the approximate beginning and end of their activities according to four categories:

- nap/sleep/rest
- work activity or leisure activity without physical effort including changing places without physical effort
- more intense physical activity, either work or sport, unusually strong emotional events. They were further instructed that the relative change between activities rather than the absoluteness of the intensity of activities should be the determining feature.

### 2.4 Analyses

**Fig 1:**
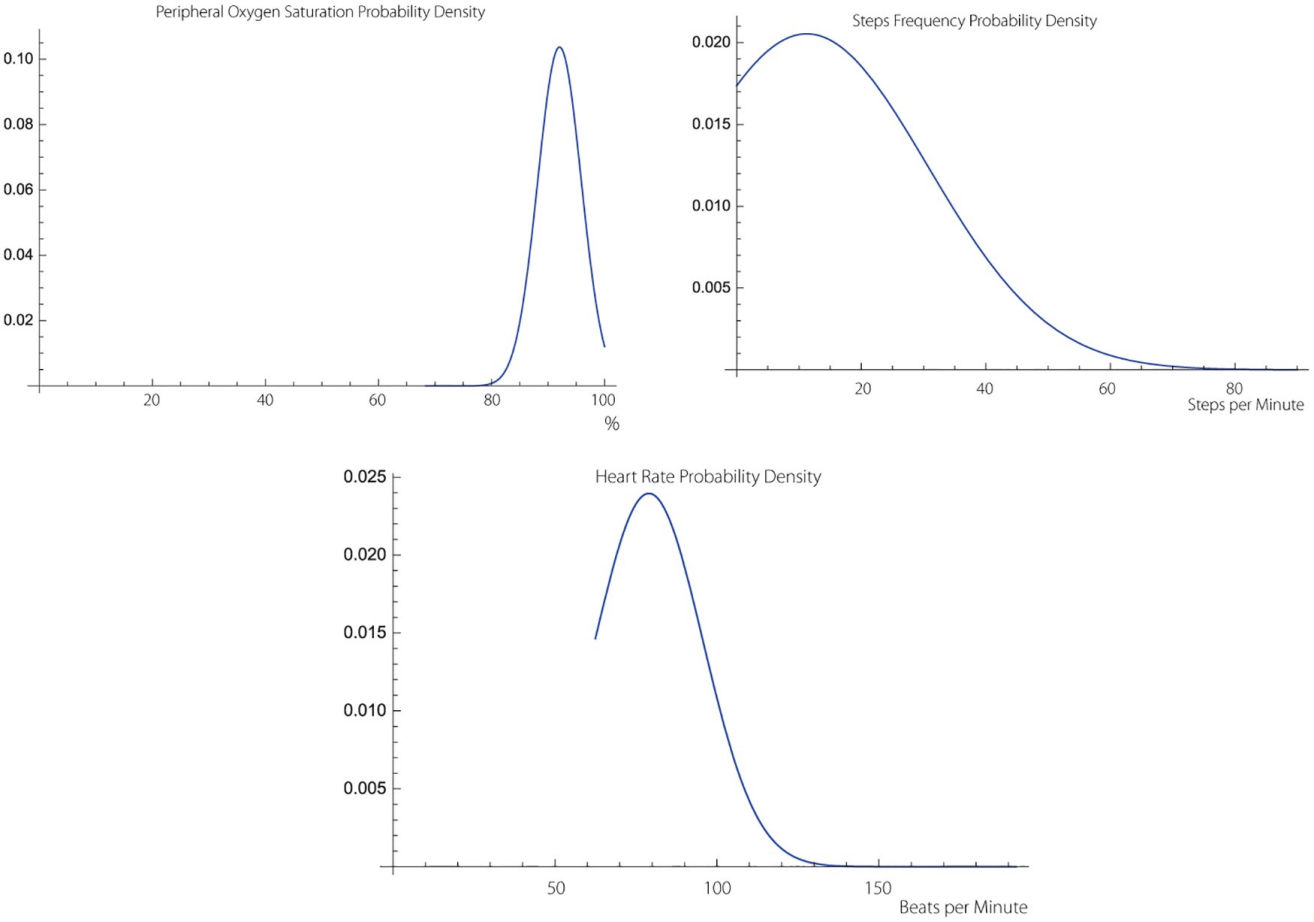
Typical probability densities of the stress indicatrix pertaining to subject 4. The densities are computed using 1, 828 data points representing 54, 850 seconds at 30 stroboscopic resolution using histograms based on 60 bins. All three densities are very close to normal distribution and possess approximate self-similarity.

We use Logical Regression (LR) [10, 11], and the geometric version on Artificial Neural Networks (ANN) [12–14], to obtain separation relative to normoxemia—hypoxemia boundaries in the *H*(*HRV*) × *H*(*SF*) complexity space S_*p*_O_2_ perceptron binary values. *H*(⋅) denotes the Hurst exponent of the enclosed SMS, [15]. We use the word “geometric” to indicate that we compute separation curves of normoxemia—hypoxemia domains. The complexity product space is based on the self-similarity scaling of normally distributed SMS time-series that can be recorded over a meso-temporal time span [16]. A typical distribution is shown at Fig 1. Complexity expressed in terms of the Hurst exponent is closely related to the Hausdorff-Besicovitch dimension.

### 2.5 S_*p*_O_2_ as a binary perceptron

The crucial choice is to select which surrogate data to consider. We choose the HRV complexity and SF complexity as the two *x* and *y* axes, which express physiological (HRV) and behavioural (SF) parameters.

We consider the pGSI to depend on three surrogate time discrete processes, i.e. HRV, SF, and S_p_O_2_. The reason for the choice is that each of them have about ten to hundred times bigger variance compared to the rest of the sensory data we measured (cf. Section 1.2 in S1 File).

Furthermore, we chose SMS with high variance that also had some degree of correlation. S1, S3 and S4 Tables in S1 File (cf. Section 1.1 in S1 File indicating correlation among HRV, SF, and S_p_O_2_). The tables indicate that SO_2_ is negatively correlated with both HRV and SF. The correlation tables also highlight the differences among different subjects.

Subsequently, we chose the complexity of S_*p*_O_2_ as the third surrogate data, Z. We turn this variable into a binary perceptron using formula (2). The perceptron provides a planar separation, given by smooth curve(s), of H(HRV) and H(SF). We lean on the following argument, tangentially supported by [17–21], leading to the choice of S_*p*_O_2_ as the binary perceptron.

*A further reason for the choice of the S*_*p*_ *O*_2_ *as binary perceptron is that stress-hormones-induced changes occur that include the CO*_2_/*pH*-*dependent decrease of the affinity of oxygen to hemoglobin due to the Bohr effect* [22], *thus increasing the oxygenation potential in the tissues.*

## 3 Results

### 3.1 The predictive Geometric Stress Index (pGSI)

We propose a view of some of the acquired SMS leading to the definition of pGSI.

Consider three time-discrete vectors $X={\left\{x\left(t_{i}\right)\right\}}_{i=1}^{n}$, $Y={\left\{y\left(t_{i}\right)\right\}}_{i=1}^{n}$, *n* ≫ 1, and $Z={\left\{z\left(t_{i}\right)\right\}}_{i=1}^{n}$ corresponding to three different sets of data representing HRV, SF and SO_2_. These quantities have different physical units and different ranges. We remove these discrepancies by projecting segmented sub-vectors on the complexity space provided by the Hurst exponent [15] or, equivalently, by the Hausdorff-Besicovitch dimension [23]. Using time equidistant coarse-grained segmentation ${\left\{t_{m}\right\}}_{m=1}^{k}$, we compute

$$\begin{array}{c} H:\left(x\left(t_{m}\right),x\left(t_{m+1}\right)\right)\mapsto \left(0,1\right],\;|\overset{k-1}{\underset{m=1}{⋃}}\left(t_{m},t_{m+1}\right)|=|t_{k}-t_{1}|,\;k>1. \end{array}$$ (1)

We compute such projections for all three quantities yielding coarse-grained complexity images of the three time-discrete vectors. We denote the new vectors by H(X), H(Y) and H(Z), respectively. We refer to the triple (H(X),H(Y),H(Z) as *stress indicatrix*. This projection, contained in (0, 1]^3^, is not invertible for we discard micro-structural information contained in the originating time-series.

Further, we construct a *binary perceptron* ${\left\{\gamma \right\}}_{m=1}^{k}$ mapping H(Z)↦{-1,1} by

$$\begin{array}{c} \gamma_{m}\overset{\text{def}}{=}\mathrm{sign}(H(\left(z\left(t_{m}\right),z\left(t_{m+1}\right)\right)-E[H(Z)]),\;\;m=1,\ldots ,k, \end{array}$$ (2)

where E[.] represents the mean of the enclosed quantity.

Considering the triples ${\left\{H{\left(X\right)}_{m},H{\left(Y\right)}_{m},\gamma_{m}\right\}}_{m=1}^{k}\in {\left(0,1\right)}^{2}\times \left\{-1,1\right\}$ we solve an optimization problem providing “optimal”, possibly closed, curve(s) defining subdomains $\Omega_{j}^{+}$ and $\Omega_{i}^{-}$, i,j∈N, of (0, 1)^2^ such that

$$\begin{array}{c} \begin{array}{cc} & \underset{i>1}{⋃}(\Omega_{i}^{+})\cup \underset{j>1}{⋃}(\Omega_{j}^{-})\\ & =(\min(H(\text{HRV})),\max(H(\text{HRV})))\times (\min(H(\text{SF})),\max(H(\text{SF}))). \end{array} \end{array}$$ (3)

The respective subdomains are convex hulls separating points $\left\{H{\left(X\right)}_{m},H{\left(Y\right)}_{m}\right\}$ with *γ*_*m*_ = 1 from the points with *γ*_*m*_ = −1. The optimization yields the smallest number of these subdomains with the largest area at the expense of allowing a small number of opposite signs to intermix, i.e., some points with *γ*_*m*_ = −1 can appear in some $\Omega_{i}^{+}$. We solve this optimization problem using a combination of untrained (geometric)ANN and LR. The optimization step yields a *stress prediction diagram* based on the complexity of the acquired SMS using geometric extrapolation that yields planar separation by a S_*p*_O_2_ binary perceptron.

Finally, we define pGSI, denoted *τ*, by

$$\begin{array}{c} \tau (H(X),H(Y);\gamma )\overset{\text{def}}{=}\frac{\mathrm{meas}({⋃}_{j\geq 1}\Omega_{j}^{+})}{\mathrm{meas}(\Omega )}\in \left[0,1\right]. \end{array}$$ (4)

### 3.2 pGSI neutrality baseline

We consider $\tau (\cdot ,\cdot ;\cdot )=\frac{1}{2}$ as the baseline. This approach is justified by the following observation. Let the underlining discrete time-series X, Y and Z be normally distributed and self-similar. Then it is plausible to assume that

$$\begin{array}{c} \begin{array}{cc} & \mathrm{meas}(\underset{j>1}{⋃}\Omega_{j}^{-})=\mathrm{meas}(\underset{i>1}{⋃}\Omega_{i}^{+})\;\text{iff}\\ & \mathrm{card}(\{\gamma_{j}=-1\;|\;j>1\})=\mathrm{card}(\{\gamma_{j}=1\;|\;j>1\}),\;\text{as}\;i,j\to +\infty . \end{array} \end{array}$$ (5)

The normality assumptions are true in our computations. Normality distribution accompanied by self-similarity of the chosen surrogate markers for stress is fundamental to characterize the complexity of SMS. The examples shown in Fig 1 are computed using a histogram map with high resolution bins.

**Fig 2:**
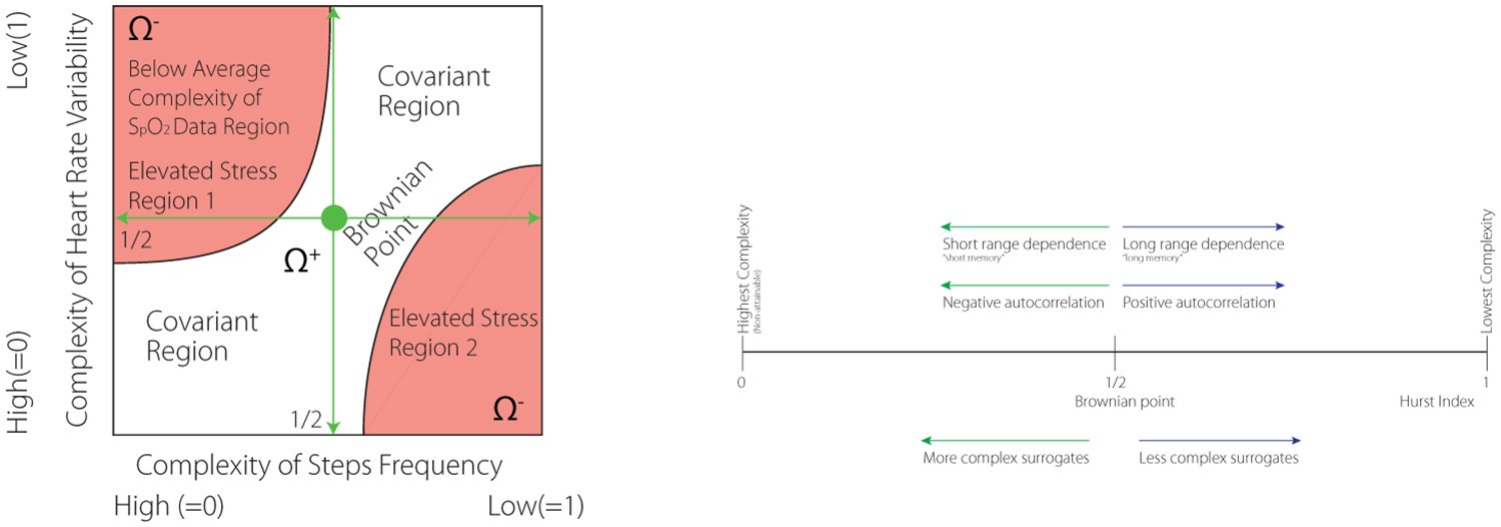
Left: Example of a segmentation of the complexity of HRV × SF space using S_*p*_O_2_ as a binary perceptron. S_*p*_O_2_ low complexity level, indicating possibly hypoxemia, accompanies incongruent complexity of HRV × SF combinations. Projecting the low levels of S_*p*_O_2_ onto the HRV × SF two-dimensional space identifies regions with undesirable HRV/SF combinations. Region 1 (cf. the left drawing) corresponds to physical stress (high SF): low S_*p*_O_2_ complexity, high SF complexity, low HRV complexity, i.e., low S_*p*_O_2_ relative to individual normhypoxemia. Region 2 corresponds to mental stress: low S_*p*_O_2_ complexity, low SF complexity, high HRV complexity, i.e., low S_*p*_O_2_ relative to individual normoxemia despite low motion levels. Right: Interpretation of the complexity indices.

The equality represents the neutral state for it equates distribution of complexities of the surrogate data. The geometry contains more information though. The *HRV* × *SF* complexity space can be divided into four subregions reflecting complexity covariance and contra-variance with respect to higher or lower than expected individual S_p_O_2_, (c.f., Fig 2). The two covariant regions, the lower-left and upper-right quadrants, share the same short/long dynamical memories as well as negative/positive autocorrelation of either complexity of HF and SF time-series. The other two quadrants have opposite characterizations. Consider the upper-left quadrant. While the *x*−axis, representing the complexity of SF, would indicate complex SMS pattern, the HRV axis indicate a more regular pattern. These readings combined with “below-the-mean” personal S_*p*_O_2_ can possibly indicate higher physical activity. Furthermore, the lower-right quadrant may indicate mental stress when the complexity of HRV and SF are reversed while the complexity of S_*p*_O_2_ is still low.

### 3.3 Predictive Stress Resistance Index (pSRI)

The predictive Stress Resistance Index (pRSI) is a further refinement of the pGSI concept. It is based on the idea that the more data points are away from the hypoxemia—normohypoxemia complexity boundary the more resistance to stress a subject will be.

The resistance index, *θ*, is defined as follows. Let

$$\begin{array}{c} m^{+}\overset{\text{def}}{=}\mathrm{card}(\{(H{\left(\text{HRV}\right)}_{j},H{\left(\text{SF}\right)}_{j})\in \underset{i>1}{⋃}\Omega_{i}^{+}\}). \end{array}$$ (6)

pSRI is then given by, c.f., the left drawing at Fig 6

$$\begin{array}{c} \theta \overset{\text{def}}{=}\frac{1}{m^{+}}\sum_{j=1}^{m^{+}}\mathrm{dist}((\{H{\left(\text{HRV}\right)}_{j},H{\left(\text{SF}\right)}_{j}\}\in \underset{i>1}{⋃}\Omega_{i}^{+}),\underset{i>1}{⋃}\partial \Omega_{i}^{-}). \end{array}$$ (7)

The pGSI is a global index. It can be achieved by uncountably many different configurations of the stress perceptrons. The predictive personalised stress diagram (pPSD) indicates desirable combinations of HRV/SF complexity configurations with respect to a higher level of S_*p*_O_2_. The red dots correspond to *γ* = −1, the green dots correspond to *γ* = 1, i.e., to normoxemia perceptrons (cf. Fig 5).

Comparing pGSI and pRSI for healthy Subject 6 (data are available upon request from authors) we conclude, as an example of the application of the pGSI/pRSI combination, that while pGSI of the Subject 6 ranks fourth. This indicates rather medium to low ability to deal with the stress, at least if our assumptions are correct (cf. Fig 7 compared to Fig 8).

### 3.4 Entropy of behavioural complexity

Consider a time discrete process $X=\{X\left(t_{i}\right),\;i=1,\ldots ,m\}$, with its complexity given by the Hurst exponent, *H*(*X*(*t*_*i*_)), computed using granulation of an underlying time-series over a uniform segmentation (*t*_*i*_, *t*_*i*+1_) of (0, *T*). The *Entropy of Behavioural Complexity* [8], is defined by

$$\begin{array}{c} bE\left(H\left(X_{m}\right)\right)\overset{\text{def}}{=}\sum_{i=1}^{m}|[\;[H(X(t_{i}))]\;]|\mathrm{sign}(H(X(t_{m}))-H(X(t_{1}))). \end{array}$$ (8)

The Hurst exponent, *H*, denotes the complexity index of acquired normally distributed SMS, ⟦⟧ denotes a jump of an enclosed quantity, i.e., $⟦\;[H(X(t_{i}))⟧\;]\overset{\text{def}}{=}H(X(t_{i}))-H(X(t_{i+1}))$, *t*_*i*_ are time equidistant points at which the function *h*: *t* ↦ *H*(*t*) has a finite jump. The signum of the difference between the complexities of previous and subsequent states indicate if the behaviours tend to a lower or higher complexity. The negative sign indicates the tendency towards higher complexity, the positive sign indicates the opposite.

We adopt the following *localized* time discrete notion of the entropy of behavioral complexity (8)

$$\begin{array}{c} bE\left(H\left(X_{m}\right)\right)\overset{\text{def}}{=}\sum_{i=1}^{m-1}|[\;[H(X(t_{i+1}))]\;]|\mathrm{sign}(H(X(t_{i+1}))-H(X(t_{i}))). \end{array}$$ (9)

The above definition of the localized entropy is a sum of signed strengths of the complexity discontinuities, (*H*(*X*(*t*_*m*_)) − *H*(*X*(*t*_1_))) is replaced by (*H*(*X*(*t*_*i*+1_)) − *H*(*X*(*t*_*i*_))).

The definition of bE, (9), accounts also for the history of attaining certain complexity states unlike its definition (8) that accounts only for the sign of the difference between initial and terminal state.

**Fig 3:**
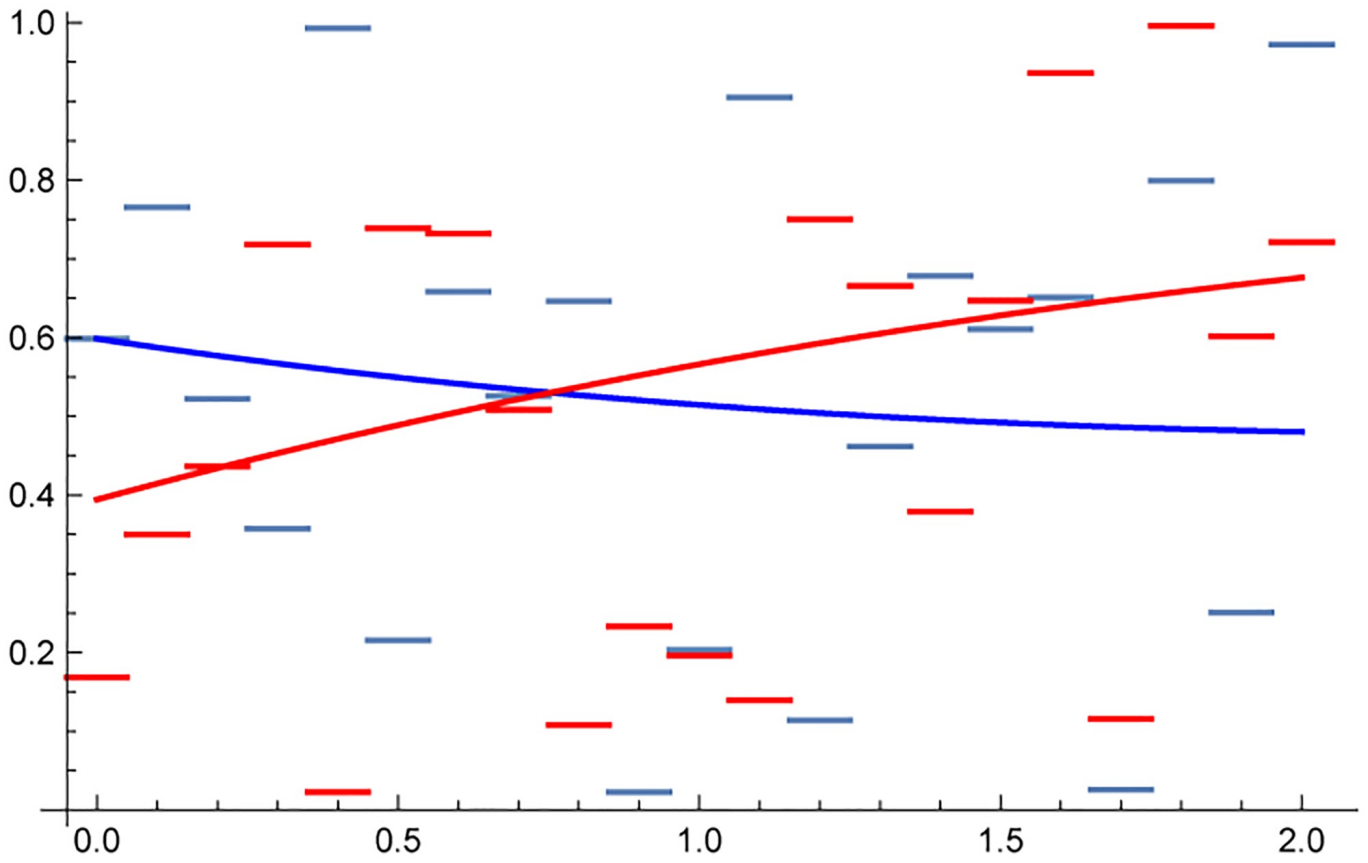
Simulated visualization of the underlying structure of the bE corresponding to two normally distributed processes yielding the two piece-wise functions simulating distribution of the Hurst exponent of a equipartitioned underlying physiological processes. The smooth curves are 4−*th* order interpolation of the given data. The negative derivative of the blue curve indicates a tendency towards more complex states with the Hurst exponent closer to zero. The red curve indicates the opposite type of behaviour. The red distribution is characterized by bE(“red”)=2.15 while bE(“blue”)=-2.95.

The idea behind the (9) definition is that bE should be negative if the system evolves, with some probability, to a state with higher complexity and positive when the system evolves towards a lesser complexity state. Let us consider the example presented in Fig 3 using synthetic data generated by normally distributed random numbers. The red piece-wise constant function is represented by bE=2.15 while the blue, decreasing function, bE=-2.95.

### 3.5 pGSI relation to bE

**Fig 4:**
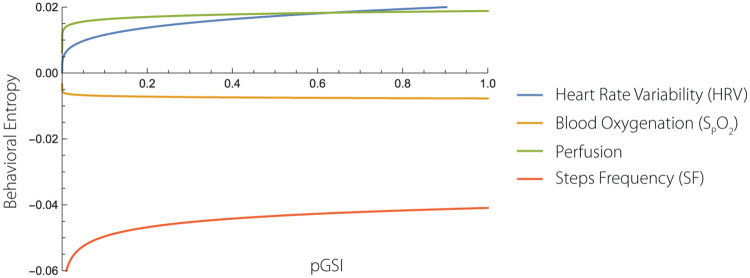
bE power laws. The figure is based on eight healthy subjects. The plot shows how the complexity of different SMS behave with the increasing stress index, pGSI. The curves show that complexity of all SMS increases except for the complexity of the S_p_O_2_.

The importance of the relationship between pGSI and bE rests with manifestation that increased levels of pGSI leads to lower levels of S_*p*_O_2_ and thus leading to increased levels of *CO*_2_ in the blood. The relationship is described below a nd visualized at Fig 4.

We propose a power law model relating pGSI to respective bE(·) having the form

$$\begin{array}{c} \alpha \;\text{pGSI}{\left(H\left(X\right),H\left(Y\right);\gamma \right)}^{\beta }∼bE\left(H\left(V\right)\right),\;\alpha ,\beta \in R, \end{array}$$ (10)

where X, Y represent HRV and SF time-series. The third quantity, Z, is represented by S_*p*_O_2_ as the binary perceptron *γ* given by (2). The time-series V represents the remaining quantities.

We identify the power law quantities, α∈R and β∈R, by solving the following non-linear problem

$$\begin{array}{cc} & \left(\alpha_{j},\beta_{j}\right)=\mathrm{Argmin}\{{\left|a\;\tau {\left(H\left(X_{j}\right),H\left(Y_{j}\right);\gamma_{j}\right)}^{b}-bE\left(V_{j}\right)\right|}^{2},\;\left\{a,b\right\}\in R^{2}\},\\ & j=1,\ldots ,\#\;\text{of}\;\text{subjects}, \end{array}$$ (11)

τ(H(X),H(Y);γ) is given by (4).

The different power laws relating pGSI to *bE* of different patterns might explain the relation between stress and complexity tendencies of SMS time-series. We include the following example as an illustration. Consider X representing HRV, Y SF complexities and Z S_p_O_2_ in the form of its binary perceptron *γ*. The computational results indicate, e.g., that

$$\begin{array}{c} 0.1\;\tau {\left(H(X),H(Y);\gamma \right)}^{0.6}\approx bE\left(H\left(X\right)\right), \end{array}$$ (12)

solving (11).

### 3.6 Power laws

The Table 1 indicates *l*_2_ distance among the real value of bE and its approximation using the presented power law (10). Scaling laws are shown in Table 2 and visualised by Fig 4.

**Table 1:** The the power law is estimated using 8 subjects.

Behavioral Entropy/Measured/Computed Quantities	HRV	Blood Oxygenation	Perfusion	Skin Temperature	Relative Movement	Steps Frequency
bE(HRV)/(Amplitude/Exponent)	0.07/ 0.4					
bE(S_p_O_2_)/(Amplitude/Exponent)		0.1/0.1				
bE(Perfusion)/(Amplitude/Exponent)			-0.0003/5.			
bE(Skin Tfemp)/(Amplitude/Exponent)				-0.2/-0.3		
bE(Movement)/(Amplitude/Exponent)					-0.2/-0.3	
bE(SF)(Amplitude/Exponent)						-0.2/0.2

The bE is computed from SMS encompassing about 15 hours of data acquisition per subject.

**Table 2:** The the power law is estimated using 8 subjects.

pGSI/Behavioral Entropy/	bE(HRV)	bE(Oxy)	bE(Perf)	bE(Temp)	bE(Mov)	bE(Steps)
pGSI(HR, SF, b-Oxy)	0.02 pGSI**0.2	-0.008 pGSI**0.05	0.02 pGSI**0.06	-0.06 pGSI**0.1	-0.09 pGSI**0.3	-0.04 pGSI**-0.08
*I*_2_ distance of scaled pGSI and bE(.)	0.114	0.117	0.215	0.309	0.385	0.271
*I*_2_ distance of pGSI and bE(.)	4.66	4.78	4.72	4.84	4.92	4.76

The behaviuoral entropy is computed from data encompassing about 15 hours of data acquisition per subject.

Fig 4 shows bE(·) of HRV, S_p_O_2_ and SF as a function of pGSI. The curves shown at Fig 4 indicate that increased level of stress leads to increased bE of HRV, Perfusion and Step frequency. The only exception to this is the complexity of S_p_O_2_ (cf. Fig 4).

### 3.7 Correlation of HRV, SF and S_p_O_2_

We use simple Pearson product-moment correlation coefficient [24], to estimate SMS dependency. The data summarised by S1-S8 Tables in Section 1.1 of S1 File show examples of correlations among different sensory quantities of three different subjects. The first two tables correspond to healthy men and women, respectively, the third table corresponds to a female marathon runner, during a typical working day including night/sleep readings. The first two tables show nearly equal negative correlation among HRV/S_p_O_2_ while that third table indicates positive HRV/S_p_O_2_ and nearly none SF/S_p_O_2_ correlations.

### 3.8 Mathematical technicalities

We address in this section some subtle points related to a construction of the personal relative hypoxemia—normoxemia domain partitions in the *H*(HRV) × *H*(SF) × *γ*(S_p_O_2_), *γ* denotes the perceptron. space, on which we can perform integration in order to compute domains areas to be able to compute pGSI. We can identify domains separation curves, we can compute distances to the separation boundaries, and we can decide which of the acquired SMS belong to which subdomain to be able to compute pSRI.

**Fig 5:**
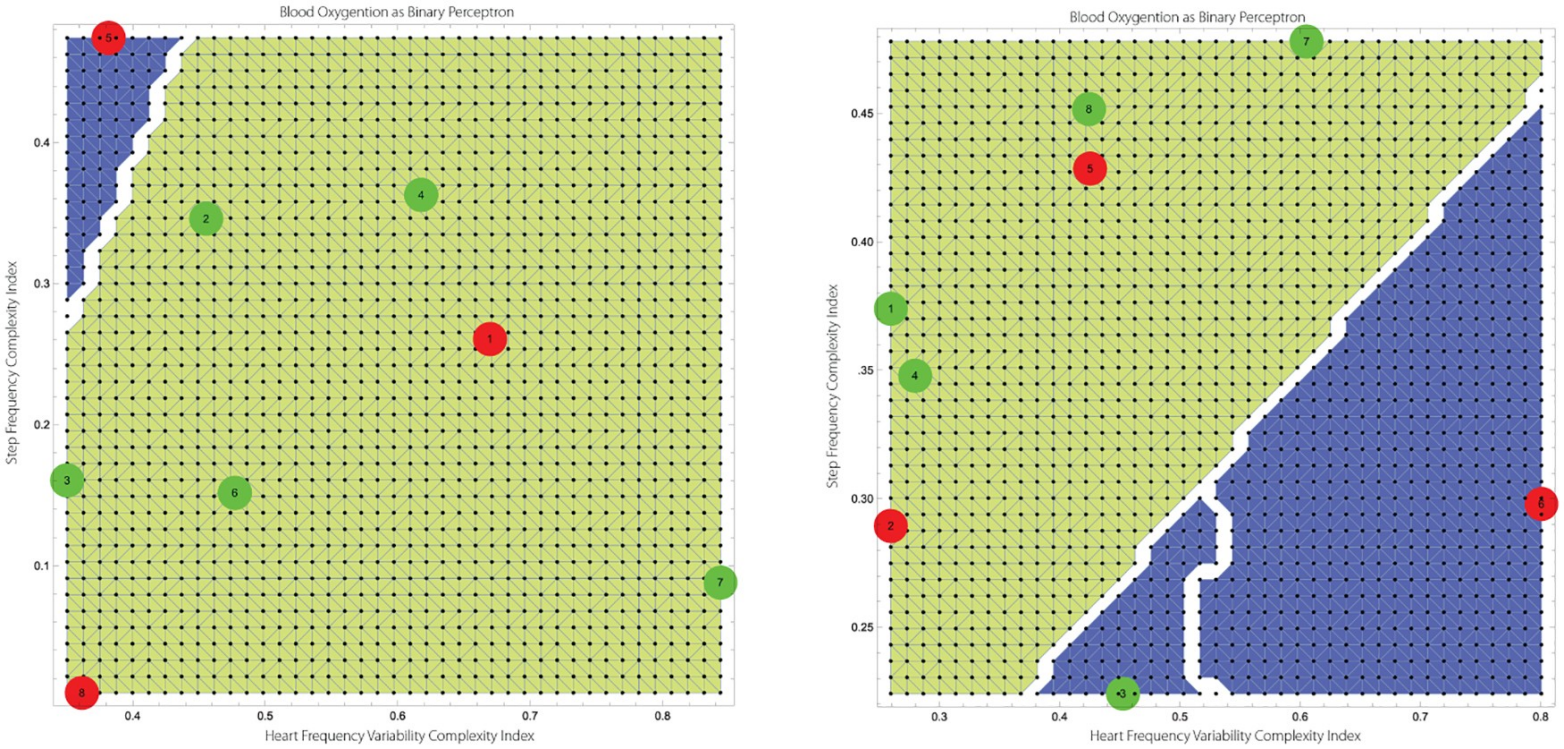
The Delaunay mesh and its separation to normoxemia and hypoxemia subdomains of subjects 4 and 6, respectively, used to compute both pGSI and pSRI. The predictive component of the analysis is associated with the assumption that the boundaries Γ_*i*_, *i* = 1, 2, should remain stable while the complexity data points can move around the effective complexity domain *H*(HRV) × *H*(SF).

We use both (geometric)ANN [25, 26] and LR [27–30], in parallel to process the complexity indices of the acquired SMS. The reason we use two different techniques is to deal more effectively with small data sets. In the next step, we apply three different techniques to identify clusters of points forming relative hypoxemia and normoxemia subdomains, i.e., Bray-Curtis Dissimilarity measure/distance (a non-Euclidian distance) [31–33], Chebyshev Distance [34], and Normalized Squared Euclidian Distance. We use Calinski-Harabasz cluster criterion, [35]. We select then the result with the least number of clusters. We then compute convexification of the respective clusters as a coarse-grained partitioning of the effective domain *H*(HRV) × *H*(SF) ⊂ (0, 1)^2^. We thus allow some hypoxemia points to belong to normoxemia subdomains and vice-versa. These steps fundamentally simplify subsequent construction of Delaunay triangulations [36, 37], based on the identified points in the complexity effective domain of the respective subdomains. Typically, we use 60 × 60 mesh points. Lastly, we disconnect the respective subdomains by small layers improving the quality of the triangulation by avoiding edges of the opposite classification. An example of the outcome of these procedures is shown in Fig 5.

### 3.9 Results pertaining to human stress and stress resistance

**Fig 6:**
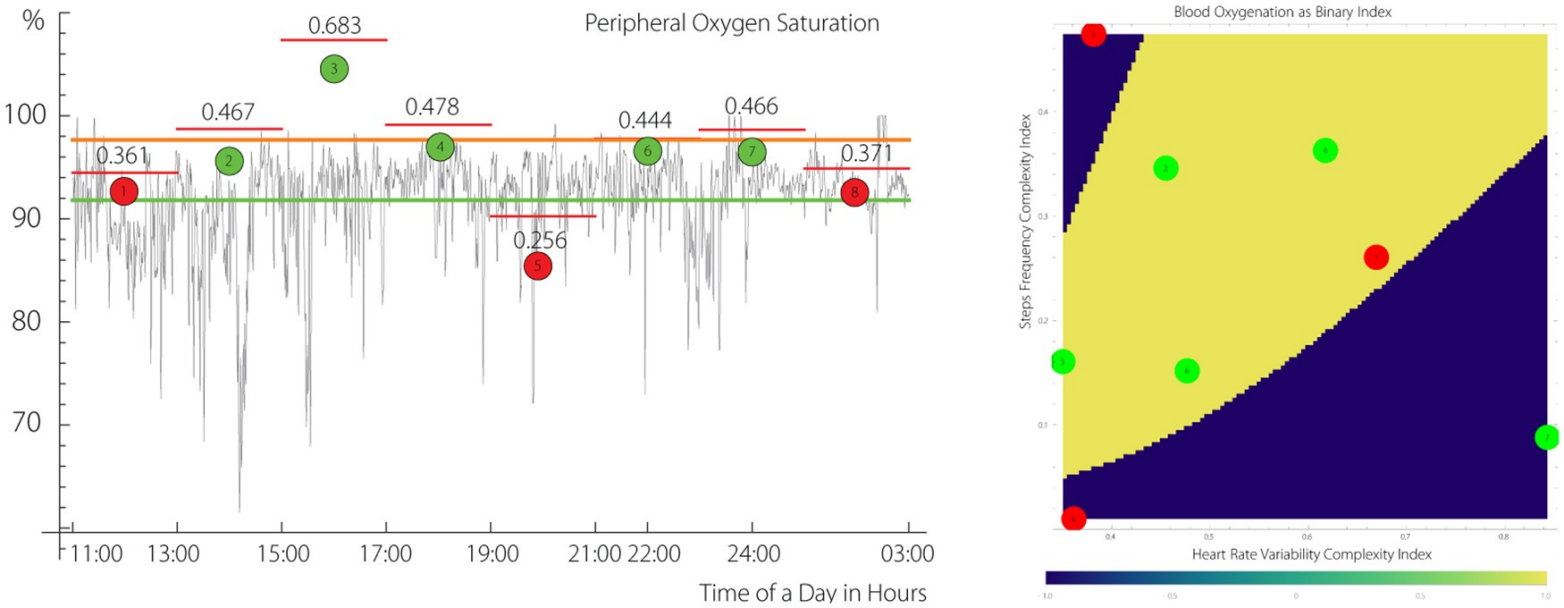
The left plot. Segmentation of S_p_O_2_) and its projection on *H*(*HRV*) × *H*(*SF*) space in the form of S_p_O_2_) using (geometric)ANN. The step-like function indicates the value of the Hurst index for each segment. *The right plot*. The yellow color indicates S_p_O_2_)-perceptron value *γ* = 1, given by (2), Ω^+^, i.e., normhypoxemia, blue colour indicates *γ* = −1, $\Omega_{i}^{-}$, *i* = 1, 2. The grey circles are positioned at the boundary of a convex hull of certain number of points with *γ* = +1. The analyzed data correspond to a human subject encapsulating 15 hours of SMS acquisition. Each segment contains about 225 data points. The green horizontal line indicates mean of S_p_O_2_), the orange represents the mean of the complexity segments.

Below we report a number of findings applying our theory to real individuals. The density plot on the right at Fig 6 shows an example indicating the personal hypoxemia(blue)—normoxemia(yellow) boundary between *H*(*HRV*) and *H*(*SF*) determined by non-linear optimization using the personal SO_2_ perceptron.

To interprete the density projection shown in Fig 6, consider two different scenarios. Focusing on the lower boundary of the normoxemia—hypoxemia domain, complexity of HRV, i.e., *H*(HRV)>1/2, exhibits lower complexity compared to SF complexity, *H*(SF)<1/2, with a ratio of approximately 1: 3. The grey point at this boundary illustrates this scenario. The combination might represent physical activity of a trained and healthy subject.

The second scenario, represented by both *H*(HRV), *H*(SF) being below 1/2, shown by the grey dot at the upper boundary of the *γ* = 1 subdomain, indicates that HRV and SF complexities approximately match. Consequently, the first scenario might correspond to a physical stress (high activity), while the second scenario corresponds to a mental stress represented by high complexity of HRV with lower complexity of SF.

Consider segment # 8 (24:00–03:00) shown at Fig 6 that corresponds to a period of sleep, in which higher complexity of HRV is accompanied by a near absence of SF complexity and a lower S_*p*_O_2_ complexity. Segment # 3 (15:00–17:00) is approximately opposite to segment # 8. The segment # 4 has all the characteristics of the first scenario, i.e. physical activity.

### 3.10 Data segmentation and coarse-graining

The data segmentation, complexity analyses results, and their projection to a three-dimensional space are shown at Fig 6.

### 3.11 pGSI and pSRI

**Fig 7:**
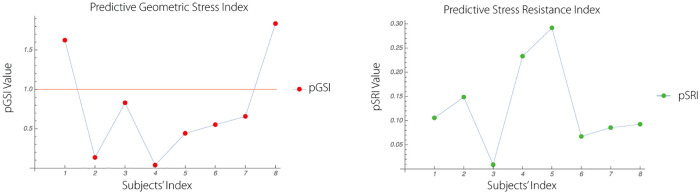
Figure shows pGSI (left graph) and pSRI (right graph) and allows comparison between the two measures. To help interpret the meaning of this comparison, we may consider two subjects as examples. Considering Subject 6 we may conclude that while his pGSI ranks fourth, his pRSI is much lower. According to our interpretation, this may indicate rather medium to low ability to deal with stress. Considering Subject # 4, we may conclude that while pGSI ranks lowest, his pRSI is among the highest of the 8 subjects. This may indicate a rather high capacity to deal with stress. Analogous interpretations may apply to the remaining subjects.

**Fig 8:**
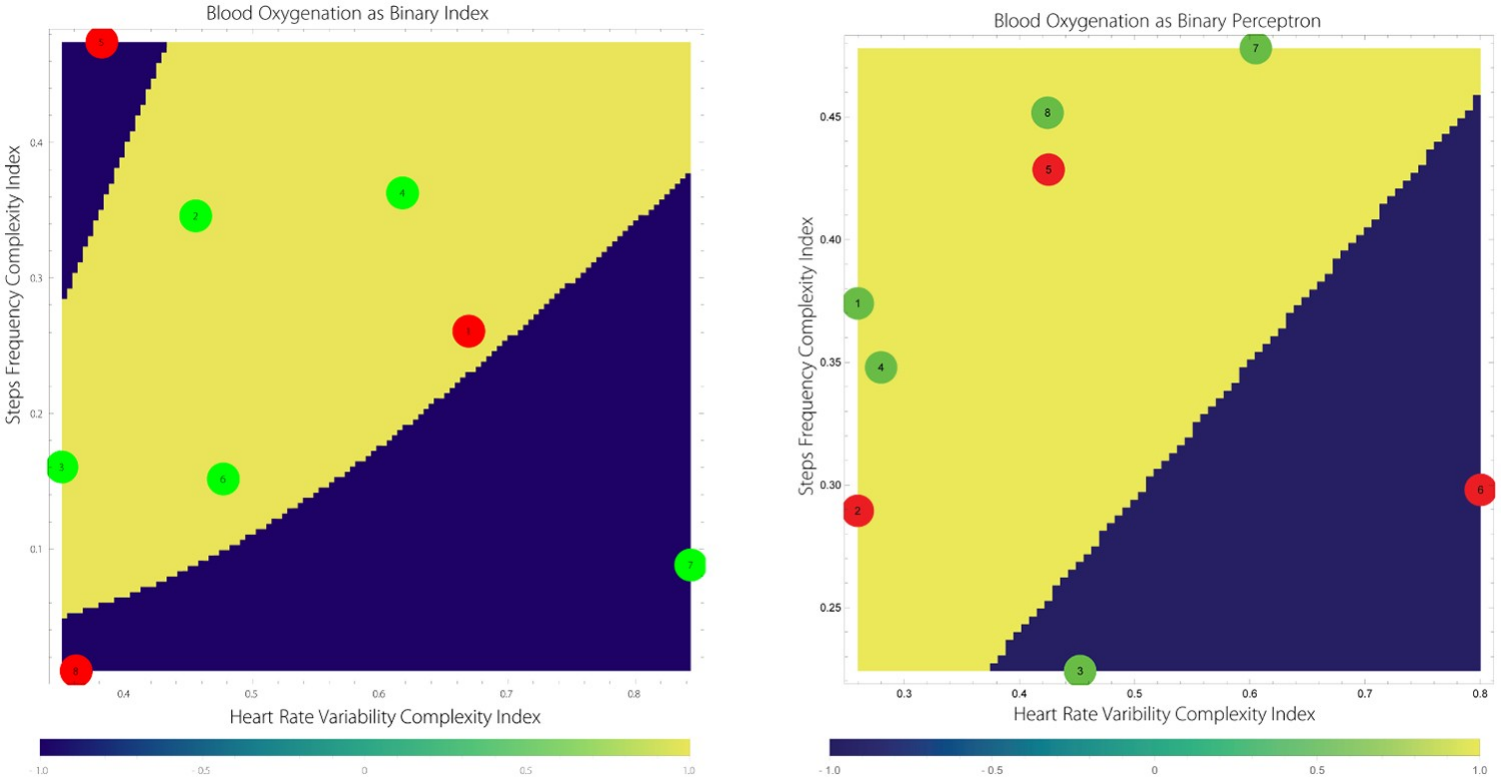
The predictive stress diagrams of subjects 4 (left) and 6 (right) shown are generated using complexity and ANN, LR analyses.

The figure Fig 8 shows comparison between the two measures, pGSI and pSRI. Comparing pGSI and pRSI for Subject 6 we conclude, as an example of the application of pGSI/pRSI combination, that while pGSI of Subject 6 ranks fourth, its pRSI is much lower. According to our interpretation, this may indicate rather medium to low ability to deal with stress (c.f., Fig 7 compared to Fig 8).

### 3.12 Recruitment

Eight healthy subjects working at SUPPA, part of the Department of Psychiatry at Lausanne University Hospital, four men and four women agreed on carrying a Biovotion’s VSM (Vital Signs Monitor) during work days including daily routines and sleep.

#### 3.12.1 Subjects’ inclusion criteria

Subjects had to be between twenty five and fifty five years and report good general subjective health.

#### 3.12.2 Subjects’ exclusion criteria

Subjects reporting the presence of any psychiatric or neurological disorders (stroke, dementia, epilepsy, tumor, schizophrenia, bipolar disorder or major recurrent depression, alcoholism or alcohol abuse or unhealthy use of other psychoactive substances.

### 3.13 Ethics

The investigation was carried under ethics application “Indexation mathématique de mesures physiologiques multiples non-invasives en milieu réel chez des sujets sains”, CHUV, Lausanne Switzerland. The ethical issues were approved by a review board at Research Ethics Commissions at the University of Lausanne, CER-UNIL.

In addition each subject signed “Informed Consent” prior to measurements of the data.

The collection and handling of data has been carried out in accordance to EU current regulations, GDPR.

## 4 Discussion and conclusion

The main idea behind our approach is to use three dimensional phase spaces to model human stress. Our approach is based on the use of S_p_O_2_ as the binary perceptron as well as HRV and SF as surrogate markers for SMS.

pGSI is derived using complexity analysis of three parameters. Namely, heart rate variability, step frequency and blood oxygenation as a binary separation parameter.

We provide an additional argument to justify our choice: the surrogate data variance. Among all the surrogate time-series the three data collections chosen exhibit the largest variance of all, c.f., S9 and S10 Tables in S1 File, corresponding to a highly trained and healthy subjects, respectively. Other data exhibit the same pattern. The combination of both the physiological and observational statistics arguments imply that the complexity characteristics of heart rate, steps frequency and S_p_O_2_ should be sufficient to index a level of stress.

The novelty of the proposed model of stress is to allow for prediction of building of a stress.

The pGSI index separates high physical activity from what we interpret as mental stress. Our analyses suggests that subjects can be distinguished regarding their overall SMS levels. Our analysis also suggests that stress is not necessarily low during sleep. Both indices, i.e., H(HRV) and H(SF) complexities correspond well with the HRV and SF raw data. Low stress modes typically exhibit a positive correlation between HRV and S_p_O_2_ while high stress modes have the opposite impact.

The results obtained using the geometric indices are very similar to those based on spectral theory [1]. However, the spectral concept is very different from the geometric one. The combination of HRV and SF complexity changes over time predicts S_p_O_2_ complexity. Based on the variable congruency between HRV and SF and the degree of S_p_O_2_ complexity, behavioural states can be extrapolated (or predicted) as either being in the normal, high-physical activity, or mental stress realm.

We will provide evidence of correlation of the results produced by our approach with those obtained through measurements of other indicators of mental stress status. Measuring subjective stress levels or dosing stress hormones in blood or saliva such as *α*-amylase, cortisol or adrenalin, as well as others are necessary to prove clinical usefulness. However, none of the measures just mentioned above can be considered absolute gold standards of stress measurements. Subjective assessment of stress may be hampered in subjects with psychiatric disorders and vary widely among the normal population. Measures of hormones or neurotransmitters in blood or saliva are necessarily coarse-grained over time as they are invasive procedures and constitute no realistic approach in clinical settings. HRV is sometimes used as another measure of stress and may be considered a gold standard for stress measures. The relationship between heart rate variability and salivary cortisol levels has been proven [5]. However, the similarity of results of our spectral and geometric approach suggests our approach is promising.

### 4.1 Context

We introduced the concept of Hausdorff-Besicovitch dimension to understand physiological and/or behavioral patterns in [8]. The idea was to coarse-grain the time-series obtained from wearable sensors and to provide dimensionless data projected on an Euclidian space. We extended this concept to human identification based on behavioral data [7] and spectral theory of stress [1] later. Our goal was to develop an equivalent to temperature pertaining to psychiatry. More importantly, our ambition in short.

Previously, we had used the concept of the Hausdorff-Besicovitch dimension to understand physiological and/or behavioral patterns in [8]. The idea was to coarse-grain the time-series obtained from wearable sensors and to provide dimensionless data projected on an Euclidian space. We extended this concept to human identification based on behavioral data [7] and provided a spectral theory of stress [1]. Our goal was to develop a simple numerical equivalent to stress, conceptually similar to what temperature is to Brownian motion.

More generally, we aim at developing, implementing and applying, within clinical environments, personalized ecological technology-assisted monitoring based on the synergistic development of novel advanced mathematical tools, novel wearable non-disruptive multi-channel biosensing, and remote diagnosis delivery mechanisms. This effort is focusing on providing objective and clinically significant diagnosis and evaluation methods for mental disorders at the behavioural level to health practitioners.

Current clinical observations and practice in mental health rely on subjective appreciations by healthcare professionals, patients and their relatives. Their accuracy critically depends on the observer’s competence and they are limited by time restraints leading to discontinued coarse-grained sampling that is often questionnaire-based, clinical in nature or occasional objective measures such as blood sampling, (f)MRI, EEG and others. More surmise that objective and continuous measures will help greatly in clinical practice to increase diagnostic efficacy, precision and, ultimately, decrease suffering and costs.

### 4.2 Limitations related to number of enrolled subjects

A major limitation of the presented paper is the small number of enrolled subjects. However, the study focus is on a new multi-dimensional model of human stress. Thus, our objective was to present this new theory as well as to validate the possibility and potential to distinguish in principle individual subjects using a limited set of selected surrogate markers for stress. For these purposes, only a small number of subjects was required. We considered a small group of subjects of whom we can precisely identify physical conditions and daily working routines. This selection then provides indirect correlation of the the theory we introduced with individual questionnaires.

However, to go beyond this proof of concept study and to demonstrate both the capacity and the usefulness of our approach to reliably monitor stress over time we will have to test it on a larger scale including more subjects, and compare it to other measures of stress such as cortisol blood measures or subjective stress levels.

## Supporting information

S1 File (PDF)

S1 Data (ZIP)
