# Supplementary material for: The geometric approach to human stress based on stress-related surrogate measures
Source: PLoS One. 2021 Jan 25;16(1):e0219414. doi: 10.1371/journal.pone.0219414 (PMC7833219; doi:10.1371/journal.pone.0219414)
Supplement: S1 File — (PDF) [file pone.0219414.s001.pdf]

S1 File

1.1 Correlation tables

Quantities	HRV	Perfusion	Blood Oxygenation	Skin Temperature	Relative Movement	Steps Frequency
HRV	1	0.312616	-0.40399	0.061539	0.620625	0.591639
Perfusion		1	-0.420654	0.0780781	0.200189	0.322364
Blood Oxygenation			1	0.00443487	-0.366836	-0.414813
Skin Temperature				1	0.0546847	0.0134603
Relative Movement					1	0.859321
Steps Frequency						1

S1 Table. The correlation of the SMS for Subject 1. The correlation was obtained from 894 equidistant time segments of acquired data.

Quantities	HRV	Perfusion	Blood Oxygenation	Skin Temperature	Relative Movement	Steps Frequency
HRV	1	-0.0144155	-0.281479	-0.178694	0.445759	0.366347
Perfusion		1	-0.0231772	-0.090395	0.103687	0.0946516
Blood Oxygenation			1	0.121536	-0.275235	-0.268467
Skin Temperature				1	-0.337818	-0.254233
Relative Movement					1	0.797544
Steps Frequency						1

S2 Table. The correlation of the SMS for subject 2. The correlation was obtained from 431 equidistant time segments of acquired data.

Quantities	HFV	Perfusion	Blood Oxygenation	Skin Temperature	Relative Movement	Steps Frequency
HFV	1	-0.0304565	-0.218817	0.101863	0.468396	0.456581
Perfusion		1	-0.0370708	0.10752	0.123471	0.143789
Blood Oxygenation			1	-0.0848257	-0.267274	-0.239368
Skin Temperature				1	0.0983426	0.0782243
Relative Movement					1	0.878678
Steps Frequency						1

S3 Table. The correlation of the SMS for subject 3. The correlation was obtained from 1160 equidistant time segments.

Quantities	HRV	Perfusion	Blood Oxygenation	Skin Temperature	Relative Movement	Steps Frequency
HRV	1	-0.17864	0.251414	0.0637194	0.625233	0.676598
Perfusion		1	-0.413867	0.0450275	0.124743	0.0341549
Blood Oxygenation			1	-0.0803077	-0.107687	-0.0287573
Skin Temperature				1	-0.0155391	0.0199515
Relative Movement					1	0.863616
Steps Frequency						1

S4 Table. The correlation of the SMS for Subject 4. The correlation was obtained from 374 equidistant time segments.

Quantities	HRV	Perfusion	Blood Oxygenation	Skin Temperature	Relative Movement	Steps Frequency
HRV	1	-0.25438	-0.286885	-0.303626	0.699491	0.580695
Perfusion		1	0.141447	0.422901	-0.044777	-0.014162
Blood Oxygenation			1	0.102708	-0.148153	-0.041951
Skin Temperature				1	-0.055175	0.0165696
Relative Movement					1	0.905904
Steps Frequency						1

S5 Table. The correlation of the SMS for subject 5. The correlation was obtained from 1826 equidistant time segments.

Quantities	HFV	Perfusion	Blood Oxygenation	Skin Temperature	Relative Movement	Steps Frequency
HFV	1	0.10924	-0.129858	0.0153075	0.350668	0.267524
Perfusion		1	-0.146234	0.112445	0.229024	0.206663
Blood Oxygenation			1	-0.187969	-0.311809	-0.207137
Skin Temperature				1	-0.0353194	-0.0188643
Relative Movement					1	0.778953
Steps Frequency						1

S6 Table. The correlation of the SMS for subject 6. The correlation was obtained from 642 equidistant time segments.

Quantities	HRV	Perfusion	Blood Oxygenation	Skin Temperature	Relative Movement	Steps Frequency
HRV	1	0.167969	-0.36686	-0.0750689	0.630799	0.608615
Perfusion		1	-0.253866	0.200398	0.284085	0.311865
Blood Oxygenation			1	0.217739	-0.437872	-0.389442
Skin Temperature				1	-0.0674336	-0.00604729
Relative Movement					1	0.93125
Steps Frequency						1

S7 Table. The correlation of the SMS for subject 7. The correlation was obtained from 1658 equidistant time segments.

Quantities	HRV	Perfusion	Blood Oxygenation	Skin Temperature	Relative Movement	Steps Frequency
HRV	1	-0.0450726	-0.112492	-0.107827	0.483597	0.479533
Perfusion		1	-0.107982	-0.0231544	-0.0138105	-0.0816
Blood Oxygenation			1	0.130734	-0.236126	-0.170944
Skin Temperature				1	-0.0825037	-0.032942
Relative Movement					1	0.901963
Steps Frequency						1

S8 Table. The correlation of the SMS for subject 8. The correlation was obtained from 452 equidistant time segments.

1.2 Two variance tables

Quantities	Segment 1	Segment 2	Segment 3	Segment 4	Segment 5	Segment 6	Segment 7	Segment 8
HRV	22.3652	37.8688	43.3020	70.4190	66.9771	20.2466	27.4566	27.9333
Perfusion	0.00210023	0.00303487	0.0142073	0.00308452	0.00448286	0.00580235	0.0437197	0.00561481
Blood Oxygenation	16.4855	44.9826	34.3267	12.5918	12.6589	19.9398	7.82296	4.68279
Skin Temperature	0.915423	0.0705794	0.018323	0.0235593	0.0229517	0.0999276	0.377792	0.27331
Relative Movement	0.522469	0.51333	1.06422	3.57759	1.56432	0.498561	0.156739	0.0188911
Steps Frequency	54.9058	30.7965	102.836	613.618	333.916	22.4165	3.5368	0.00266509

S9 Table. The variance of the SMS for Subject 4. The variance was obtained from 374 equidistant time segments of acquired data.

Quantities	Segment 1	Segment 2	Segment 3	Segment 4	Segment 5	Segment 6	Segment 7	Segment 8
HRV	339.983	101.478	118.456	146.107	522.311	34.4726	19.3977	13.9784
Perfusion	0.0543121	0.0315185	0.00918733	0.00217061	0.0114509	0.00109113	0.00178169	0.0134619
Blood Oxygenation	66.8304	68.556	16.1578	13.3593	12.07	2.83156	3.41918	8.13148
Skin Temperature	0.425757	0.44536	0.488427	0.197185	2.53583	0.2535	0.350932	0.0978087
Relative Movement	4.34773	0.263877	3.22692	2.39485	5.16624	0.251206	0.31262	0.712321
Steps Frequency	447.909	20.6665	356.858	216.062	772.406	4.34818	10.0686	22.1422

S10 Table. The variance of the SMS for Subject 6. The variance was obtained from 642 equidistant time segments of acquired data encompassing about 15 hours of data acquisition.
